# Welcome to *International Journal of Retina and Vitreous*

**DOI:** 10.1186/s40942-015-0004-9

**Published:** 2015-04-15

**Authors:** Eduardo B Rodrigues, Jay S Duker, André V Gomes

**Affiliations:** 1 Federal University of São Paulo, Rua Botucatu, 821, São Paulo, SP Brazil; 2 New England Eye Center, 260 Tremont St, Boston, MA USA; 3 Brazilian Society of Retina and Vitreous, Alameda Santos 1343 sala 408, São Paulo, SP Brazil

It is our great pleasure to introduce a new publication devoted exclusively to diseases of the posterior segment of the eye *- International Journal of Retina and Vitreous* (IJRV). The launch of this new journal is the natural outgrowth of several recent macro trends in our specialty.

Since its creation as a subspecialty more than 50 years ago [1], the field of retina-vitreous has undergone more revolutionary changes in the past decade than any other in medicine. Our ability to not only diagnose diseases with incredible accuracy in the office with advanced multi-modal imaging, but also our improvement in the ability to treat diseases in both the office and the operating room is truly remarkable. In this context, the increase in the numbers of specialists and researchers concentrating on the posterior segment has generated a growing number of scientific articles in this field. In 2013 alone more than 3000 articles were published related to retinal research. Wide dissemination of knowledge permits both further scientific gains and practical clinical results - and this progression exemplifies the niche of journal like IJRV.

The goal of IJRV is to provide up-to-date, accurate, and easily understandable information to our readership and to contribute to the body of scholarly work in the area of retina and vitreous. The journal will present original articles on new approaches to diagnosis and treatment that may impact clinical practice. As part of this vision, our aim is for IJRV to focus on articles that directly impact the clinician’s ability to treat patients, and will include special topics of interest such as imaging of the retina, choroid and vitreous especially innovations in optical coherence tomography (OCT) and other imaging systems, small-gauge vitrectomy, retinal detachment repair, chromovitrectomy, electroretinography, microperimetry, other functional tests, intraocular tumors, retinal pharmacotherapy & drug delivery, diabetic retinopathy & other vascular diseases and age-related macular degeneration & other macular entities [2–5].

The peer-review process involves two Editors-in-Chief, seven Associate Editors, and a Board composed of over 50 outstanding international experts in the field of retina [6]. All manuscripts will be peer-reviewed, and we are fortunate and honored to have recruited an Editorial Board of recognized experts in their field to help with this process.

## Journals in Retina

For the science of retina, existing journals serve the field very well and published important research. We hope to supplement the existing journals with a different format. Our aim is to bring together diverse areas of research in retina and vitreous with three novel approaches. Firstly, IJRV will be entirely web based with paper based copies only by request, although PDF of articles will be available through the website. Secondly, IJRV will be an open access journal, which means access to its content with no subscription barriers. Third, initially we will be fortunate to be supported by the Brazilian Retina and Vitreous Society, which is covering the costs of publication so that authors do not need to pay an article processing charge

With these online, open access characteristics, IJRV intends to allow timely publication and broad dissemination of research to a global audience of ophthalmologists, retinal specialists, retina-interested physicians, and trainee physicians. As electronic and digital communication allows rapid exchange of ideas and information, while discoveries are made continuously and following experimentation and analysis, research should be disseminated immediately [7]. With the internet, this has become possible and easier. Various recent publications report that articles in open access journals generate more citations, more downloads and reach a broader audience within the first year [8, 9].

## Brazilian Retina and Vitreous Society

While this journal expects to be one of the pre-eminent international publications, it also serves as the representative journal for the Brazilian Retina and Vitreous Society. When the Brazilian ophthalmologists Joviano Rezende Filho, Luiz Assumpção Osório, Francisco Mais, Sérgio Cunha, and Christiano Barsante met in September 7th 1977, they knew the field of retina deserved a new society to enable discussion and scientific progress in retina. At that time, they organized the first Brazilian retinal meeting - a course with Prof. Charles Schepens that involved around 250 ophthalmologists in Rio de Janeiro. From there the BRVS was founded in 1977 with the mission to stimulate discussion and scientific progress in retina in Brazil. BRVS is now composed of around 900 dedicated active members fascinated by the field of retina and vitreous.

Since its inception, the BRVS and its members have actively participated in international symposia and published scientific articles in important medical journals thereby assisting in the progress of the area of research. For the past few years many BRVS members have worked very hard to enable development of a new Journal and to insert the society in the global network of retinal research. This effort resulted in the partnership with BioMed Central to launch IJRV. The BRVS and its partners are also proud to join the open access era by publishing a journal for the great field of retina.

## Conclusions

In summary, we hope that IJRV will serve as a vehicle for bringing together multidisciplinary perspectives, research and initiatives from around the globe who are interested in advancing the field of retina. We would welcome your comments on the articles and other work published in the journal. As Co-Editors-in-Chief, together with our international Editorial Board, we are committed to making this journal a success, and your support is welcome and appreciated.

IJRV realizes the importance of indexing databases (e.g. PubMed, Web of Science, Scopus and Medline) and will have the Journal submitted to those services as soon as possible. We intend to have the journal registered with the Directory of Open Access Journals, and cited by PubMed and other search engines in the very near future. On behalf of the Editors and Editorial Board members, welcome to the *International Journal of Retina and Vitreous*.
